# IL12 p35 and p40 subunit genes administered as pPAL plasmid constructs do not improve protection of pPAL-LACK vaccine against canine leishmaniasis

**DOI:** 10.1371/journal.pone.0212136

**Published:** 2019-02-22

**Authors:** Pedro J. Alcolea, Ana Alonso, Adriana Esteban, Paz Peris, Alberto Cortés, Juan A. Castillo, Vicente Larraga

**Affiliations:** 1 Laboratory of Molecular Parasitology, Department of Cellular and Molecular Biology, Centro de Investigaciones Biológicas, Consejo Superior de Investigaciones Científicas, Madrid, Spain; 2 Laboratory of Parasitology, Department of Pathology, Facultad de Veterinaria, Universidad de Zaragoza, Zaragoza, Spain; Academic Medical Centre, NETHERLANDS

## Abstract

*Leishmania infantum* causes zoonotic visceral leishmaniasis (ZVL) in the Mediterranean basin and South America. The parasite has been shown to co-infect HIV patients and an outbreak in central Spain was reported in the last decade. Therfore, ZVL is a public health problem, dogs being the parasite's reservoir. We have developed a DNA vaccine based on the *L*. *infantum* activated protein kinase A receptor (LACK) using different plasmid vectors and vaccinia virus strains as vehicles. Recently, we have generated an antibiotic resistance marker-free plasmid vector called pPAL. Homologous pPAL-LACK prime-boost vaccination protects Beagle dogs as well as a heterologous plasmid-virus regime. For both reasons, pPAL improves safety. IL12 was described to trigger Th1 response through IFN-γ production in infected dogs, being a good candidate for cytokine therapy in conventional treatment-unresponsive dogs. Herein, we report a complete protection study in dogs through inoculation of genes encoding for the p35 and p40 subunits which compose canine IL12 in combination with the LACK gene. A homologous plasmid-plasmid regime using independent pPAL constructs for each gene was inoculated in a 15-day interval. The infectious challenge using *L*. *infantum* promastigotes was successful. The outcome was pPAL-LACK vaccine protection suppression by IL12 administration. The important implications of this finding are discussed in the manuscript.

## Introduction

Leishmaniasis is a disease caused by trypanosomatid parasites grouped into the genus *Leishmania*. Human leishmaniasis is classified according to the clinical manifestations into cutaneous (CL), mucocutaneous (MCL), and visceral leishmaniasis (VL). VL by *Leishmania infantum* is a zoonosis (ZVL), whereas VL by *Leishmania donovani* is anthroponotic. VL is fatal without treatment and causes about 50,000 annual deaths worldwide [1]. HIV-*Leishmania* co-infections have been reported [2,3,4,5,6]. Canids are *L*. *infantum* (syn. *L*. *chagasi*) reservoirs in the Mediterranean basin, Central Asia and South America [7,8]. An *L*. *infantum* outbreak has been registered in humans during the last decade in central Spain [9,10,11,12,13,14]. As a difference with human VL, variable cutaneous and visceral clinical signs simultaneously occur in dogs depending on the individual. The clinical profiles range from asymptomatic to severe, including weight loss, asthenia, cutaneous lesions, conjunctivitis, anorexia, lymphadenopathy, hepatomegaly, splenomegaly, etc. [15,16,17]. Bone marrow may become densely parasitized in infected dogs, causing histiocytic hyperplasia, erythrocytic hypoplasia, granulomas, and megakaryocytic emperipolesis and dysplasia [18].

*Leishmania* spp. parasites develop a digenetic life cycle defined by two stages. Promastigotes are fusiform motile cells with a flagellum emerging from the cellular body anterior pole. This stage differentiates in the gut of sand flies (Psychodidae: Phlebotominae) from procyclic to highly infective metacyclic forms. Simultaneously, promastigotes migrate towards the anterior gut. Metacyclic promastigotes are inoculated in the mammalian host's dermis during bloodmeal intakes. A few promastigotes not cleared by the complement system are internalized by phagocytic mononuclear cells, where they differentiate to the obligate intracellular amastigote stage and multiply inside phagolysosomes. Amastigotes are released when the host cell colapses and are able to infect other phagocytes. A sand fly becomes infected when feeding from an infected mammal's blood (reviewed by Moody [19]). The immune response against *Leishmania* spp. is partially understood only in the mouse-*L*.*major* infection model. In this case, lymphocyte T helper response type 1 (Th1) leads to protection, whereas Th2 leads to susceptibility [20]. Interleukin 10 (IL10) is able to inhibit interferon-γ (IFN-γ) and IL12 production, leading to susceptibility [21]. IL12 is a proinflammatory cytokine against intracellular pathogens. The Th1/Th2 dichotomy is absent in canine leishmaniasis and human CL and VL. For example, IFN-γ decreases in humans at the early CL infection stages [22], but gradual IFN-γ and tumoral necrosis factor α (TNF-α) increase is observed, leading to self-healing over time [21]. A balanced Th1/Th2 response seems to trigger protection against *L*. *infantum* in dogs, as suggested by γ-IFN and IL10 patterns [23,24,25,26,27]. IL12 is able to trigger IFN-γ production in peripheral blood mononuclear cells (PBMC) of *L*. *infantum* infected dogs [28]. For this reason, treatment with IL12 may lead to healing.

The *L*.*infantum* activated protein kinase A receptor analogue (LACK) is located in the cytoplasm particulate fraction [29], and is able to protect BALB/c mice against *L*. *major* [30,31] and *L*. *infantum* [32], and Beagle dogs against *L*. *infantum* [27,33,34] experimental infection when administered as a DNA vaccine. The antibiotic resistance marker-free mammalian expression plasmid vector pPAL has been used as a new vaccination vehicle for the LACK gene. The pPAL-LACK vaccine is approximately as efficacious against canine leishmaniasis when administered following a plasmid-plasmid homologous regime as when an heterologous plasmid-recombinant vaccinia virus regime is followed [26]. Efficacy is higher by the intranasal than by the subcutaneous route in mice [35] and in dogs [26]. The aim of the present study is evaluating the efficacy of canine IL12 subunit genes as possible adjuvants of the pPAL-LACK vaccine.

## Methods

### Parasites and antigens

A bone marrow explant from a naturally infected retriever dog was cultured at 26°C in NNN (Novy–Nicolle–McNeal) medium in order to axenize *L*. *infantum* promastigotes (isolate MCAN/ES/MON1/Z001), which hold genotype A and zymodeme MON-1 (Maribel Jiménez and Ricardo Molina, personal communication) according to ITS- [36] and CPB-based [37] genotyping and zymodeme determination [38,39]. Promastigotes were transferred to complete medium (CM) composed of RPMI 1640 supplemented with 2mM glutamine (Gibco BRL) and 10% of heat-inactivated (56°C, 1h) fetal bovine serum (Cambrex). 100μg of streptomycin and 100IU penicillin/ml (Gibco BRL) were added to the culture media. Stationary phase (day 7) promastigotes were obtained for challenge four passages after bone marrow explant cultivation.

Crude *L*. *infantum* antigen (CLA) was obtained from 8x10^8^ cell/ml stationary phase promastigote suspension in PBS applying three freezing-thawing cycles (-20°C/room temperature). Soluble leishmania antigen (SLA) was prepared by CLA sonication followed by centrifugation at 16,000x*g* for 3min. Recombinant LACK protein was expressed in heterologous system and purified as follows using a pRSETB-LACK *E*. *coli* BL21 pLysS clone obtained by González-Aseguinolaza et al. [29]: i) growth of a 20mL inoculum at 37°C for 16h in liquid LB medium containing 100μg/mL ampicillin; ii) dilution to 1L LB-ampicillin and growth until reaching 0.5 OD_600nm_; iii) 1h induction with 1M IPTG; iv) centrifugation at 4,000g for 20 min; v) pellet re-suspension and mild agitation in 10mL of buffer A (6M guanidine hydrochloride, 0.1M NaH_2_PO_4_, and 10mM Tris-HCl, pH 8.0; vi) centrifugation at 10,000x*g* for 30min; vii) equilibration of 3mL Ni-NTA resin (Qiagen) with 10mL buffer A; viii) batch incubation of the resin with the supernatant obtained in step vi for 1h at room temperature; ix) resin packaging on column and lysate elution; x) wash with 8mL buffer B (8M urea, 0.1M NaH_2_PO_4_, and 10mM Tris-HCl, pH 8.0); xi) two additional wash steps with 4mL buffer C (8M urea, 0.1M NaH_2_PO_4_, and 10mM Tris-HCl, pH 6.3); xii) elution with 10mL buffer D (8M urea, 0.1M NaH_2_PO_4_, and 10mM Tris-HCl, pH 5.9); xiii) elution with 10 ml buffer E (8M urea, 0.1M NaH_2_PO_4_, and 10mM Tris-HCl, pH 4.5). In all elution steps, 2.5mL fractions were collected. All steps were analyzed by 12% SDS-PAGE. For this purpose, 0.1% uninduced/induced culture volume aliquots were centrifuged at 13,000x*g* for 1min and resuspended in 30μL Laemmli loading buffer; or 20μL eluate aliquots were taken, diluted to 180μL with PBS, precipitated at 4°C for 15min with trichloroacetic acid (adding 20μL of a 50% stock solution), centrifuged at 15,000g for 15 min, washed with 200μL pre-cooled absolute ethanol, air-dried, and resuspended in Laemmli loading buffer. Purified LACK protein fractions were dialyzed three times against PBS (30min each), and finally against 0.1% SDS in PBS for 16h. CLA, SLA, and LACK antigens were quantified by the Bradford method.

### pPAL-LACK, pPAL-IL-12p35 and pPAL-IL-12p40 plasmid constructs

The pPAL-LACK third generation vaccine was generated as described [26,40]. Canine IL12-p35 and p40 subunit gene ORFs (GenBank accession numbers U49085 and U49100) flanked by appropriate restriction sites were chemically synthesized and delivered in pGH plasmid vector (ATG Biosynthetics): MluI site in the 5’ end of both genes, SalI site in the canIL12-p35 3’ end, and XbaI site in the canIL12-p40 3’end. *E*. *coli* XL1-blue competent cells were prepared following the procedure described by Hannahan et al. [41] and each 100μL cell aliquot transformed with 10μL of a 0.5ng/μL plasmid solution by a heat shock procedure as follows: i) 4°C for 20min; ii) 37°C for 1min; iii) 4°C for 2min; iv) 37°C for 1h after dilution with LB liquid medium. 10% of the transformation mix volume was plated onto LB-agar containing 100μg/mL ampicillin. Positive clones of each construct were then expanded at 37°C for 16h in 20ml liquid LB medium in the presence of 100μg/ml ampicillin and purified with High Pure Plasmid Isolation Kit (Roche) following the manufacturer's instructions.

pGH-canIL12-p35 plasmid was digested with MluI/SalI at 37°C for 1 h using NEB3 buffer and 0.1mg/mL BSA. The same conditions were applied to digest pPAL with MluI/SalI. The desired digests were isolated by 1% agarose gel electrophoresis. The bands were excised and purified with QIAquick Gel Extraction Kit (Qiagen), and the 50μL eluate was diluted to 100μL with milliQ water and precipitated with 0.1 volume of 3M sodium acetate and 2.5 volumes of pre-chilled absolute ethanol at -20°C for 30 min. The mixture was centrifuged at 15,000x*g* for 20min and the pellet washed with 70% ethanol, air dried and resuspended in 25μl of milliQ water. The same digestion and purification process was followed for pGH-canIL12-p40 and pPAL using only MluI in NEB3 buffer because MluI and XbaI are not compatible for digestion in the same NEB buffer. Once pGH-IL12-p40 and pPAL MluI digests were purified, the entire process was repeated with XbaI. Inserts and vectors were quantified by 260nm UV spectrophotometry and densitometry of 1% agarose gel electrophoretic runs. The ligation reactions of insert with 50ng vector at a 5:1 molar ratio were performed at room temperature for 1h using 400 Weiss units of T4-DNA ligase and buffer (NEB) in a final volume of 10μL. Ligation mixtures were used to transform the *E*. *coli* SURE strain (Agilent Technologies) following the manufacturer's instructions. 10% of total transformation mixture volumes were plated onto LB-agar containing 5μM triclosan and grown at 37°C for 16h. Clone expansion and plasmid isolation was performed as specified above. Recombinant clones were selected by 1% agarose gel electrophoresis analysis and confirmation was performed by Sanger sequencing. A selected recombinant clone of each construct (pPAL-canIL12-p35 and pPAL-canIL12-p40) was grown in the presence of 5μM triclosan and purified using Endotoxin-Free Qiagen Plasmid Mega Kit (Qiagen). Plasmid quantification was performed as specified above. pPAL-canIL12-p35 and p40 plasmid maps and sequences are shown in **S1** and **S2 Figs**.

### Animals, ethical statements, plasmid inoculation and infectious challenge

All measures available to ameliorate suffering of five female and five male 12-18-month old ~13 Kg beagle dogs (CEDS) used in the experiment were taken. Dogs were lodged, maintained, and used for experimentation at optimal conditions: temperature control, ad-libitum feeding, procedure refinement, disease monitoring, behavioral enrichment, daily controlled release outdoors, etc. Experimental design and procedures included in project [Safety, immunogenicity, and efficacy study of the DNA vaccine pPAL-LACK after challenge with *Leishmania infantum* in dogs] were approved by the University of Zaragoza Ethical Advisory Commission for Animal Experimentation (11/03/2010, reference PI12/10). Two animal groups were set: i) group 1 (GI), PBS-dissolved 200μg pPAL-LACK + 20μg pPAL-canIL12-p35 + 20μg pPAL-canIL12-p40 prime-boost inoculation by the intranasal route (60 and 45 days before challenge); and ii) challenged control group (group 2, GII). Challenge was performed by the i.v. route with 10^8^ *L*. *infantum* stationary phase promastigotes. 2-mL blood samples were taken 60 days before and 40 days after experimental infectious challenge (-60 dpi and 40dpi) for humoral response analysis. 10-mL blood samples were extracted -7, 21, 180, 240, and 300 dpi for humoral and cellular immune response analysis. 100-μL bone marrow samples were obtained 0, 21, 180, and 240 dpi for parasite burden analysis. At the end of the study (300dpi), dogs were euthanized using 0.3ml/kg T61 (Intervet) by the intravenous route. Liver, lymph node, bone marrow, and spleen samples were collected during necropsy for parasite burden and cellular immune response analysis.

### Evaluation of clinical signs

Canine leishmaniasis clinical signs (alopecia, anemia, diarrhea, exfoliative dermatitis, epistaxis, hepatomegaly, splenomegaly, keratoconjunctivitis, lethargy, lymphadenopathy, muscular atrophy, onychogryphosis, seborrheic dermatitis, skin ulcers, weight loss, etc.) were registered in clinical record forms. Intensity of every clinical sign was scored in a natural number scale ranging from 0 to 5. Numerical evaluation of clinical signs (NECS) is an approximation to illustrate the overall dog clinical profile which is calculated as the sum of all scores registered in an animal.

### Parasite burden evaluation

Genomic DNA was isolated from bone marrow aspirates using NucleoSpin® Blood (Macherey-Nagel) following the manufacturer's instructions. Francino et al. [42] LEISH-1 primer (AACTTTTCTGGTCCTCCGGGTAG), LEISH-2 primer (ACCCCCAGTTTCCCGCC), and LEISH-P TaqMan MGB probe (6-FAM-AAAAATGGGTGCAGAAAT) distributed onto 384-well plates using the Assays-by-Design service (Applied Biosystems, Life Technologies) were used for quantitative parasite burden evaluation. Reactions were performed in a 7900HT Fast Real Time PCR system (Life Technologies) using 2x TaqMan® Universal Master Mix (Life Technologies) and genomic DNA template 1:5 serial dilutions (1, 0.2, and 0.04ng/μl) in a final volume of 10μL. Thermal cycling was carried out as follows: 95°C for 5min; 40x[95°C for 30s; 60°C for 1min, data acquisition]. Two technical replicates per sample were performed. Eukaryotic 18S rRNA Pre-Developed Taqman Assay Reagents (Applied Biosystems, Life Technologies) were used as endogenous reference performing the same sample dilutions and technical replicates. Precautions to avoid contamination with external DNA were taken at all times throughout the experiments, including separate reagent and sample manipulation and storing locations, as well as surface decontamination using 10% bleach. Coefficients of variation were checked (≤20%), and PCR efficiencies were calculated using the linear regression method. Raw quantities are efficiencies to the power of negative Ct. Normalized quantities were obtained by dividing LEISH by 18S rRNA quantities. Best fit equation obtained from linear regression of absolute quantification standards was applied to calculate the number of parasites in 10ng sample. The number of amastigotes per mg tissue was estimated using a correction factor based on genomic DNA purification yield.

### Humoral immune response assessment

2-mL peripheral blood collected from dogs were allowed to clot at room temperature for 20min using Eurotubo® Serum Separation polypropylene tubes (Deltalab) with accelerant and granules, and centrifuged at 1,000x*g* at room temperature for 10min. Serum was stored at -20°C and used for ELISA assessment of total IgG, IgG1 and IgG2 levels. First, 96-well flat-bottom microplates were coated with 100μl of 10μg/mL SLA in 3.36mM carbonate– 10mM bicarbonate buffer at 4°C overnight, blocked with 1% BSA in PBS, and washed three times with 0.1% BSA, 0.03% Tween 20 in PBS. Next, 100μL of 1:10 serum sample was added to each well and allowed at room temperature for 30min. Meanwhile, HRP-conjugated anti-dog IgG1 and anti-dog IgG2 antibodies (Bethyl Laboratories) were respectively diluted 1:15,000 and 1:20,000 in blocking solution, and protein A (Sigma-Aldrich) was prepared at 0,19μg/mL using washing solution. Samples were washed before and after incubation with 100μL of the corresponding antibody or protein A solution at room temperature for 2h, although the last washing step was carried out with PBS. An O-phenylendiamine dihydrochloride (OPD) tablet was dissolved in 12mL of citrate buffer (5.2mM citric acid, 12.7mM Na_2_HPO_4_, pH 5.0) and 15μL H_2_O_2_ were added. Color development was allowed for 20min after adding 100μL of this solution to each well. The reactions were stopped by adding 50μL of 1% SDS, and absorbance was read at 450nm using Microplate Reader 680 (BioRad) and Microplate Manager 5.2.1. software (BioRad).

### Lymphoblastic transformation test (LTT)

Peripheral blood samples (8mL) were collected in lithium-heparin BD Vacutainer® tubes (BD Biosciences). Once blood samples were diluted with 10mL PBS, peripheral blood mononuclear cells (PBMC) were isolated by centrifugation over 7mL Lymphoprep^TM^ (1.077g/mL, Axis-Shield) at 750x*g* (no brake) at room temperature for 30min. Target organ (liver, lymph node, or spleen) ~0.5cm^3^ portions were disaggregated using BD Falcon Cell Strainers (BD Biosciences) Buffy coats or target organ cell suspensions were washed in 50mL PBS, incubated in 1mL erythrocyte lysis solution (0.15M NH_4_Cl, 10mM KHCO_3_, 0.1mM EDTA, pH 7.2) for 10min, labelled with 10μg/mL carboxyfluorescein succinimidyl ester (CFSE, Molecular Probes) during 20min, washed with RPMI twice, and cultured at 37°C in the presence of 5% CO_2_ for 3 days in CM containing 50μg of β-mercaptoethanol in the presence of different stimuli (1μg/mL concanavalin A, 10μg/mL denatured recombinant LACK protein, or no stimulus). Then, cells were harvested in 96-deep-well plates (Eppendorf), supernatants stored at -20°C and pellets washed twice in 1% BSA in PBS, blocked in the same solution for 1h, and incubated with ~0.1mg/mL R-phycoerythrin (RPE)-conjugated rat anti-canine CD4 (Serotec), once each vial’s content had been dissolved in 1 ml milliQ water and this solution 1:10 diluted using 1% BSA in PBS. Next, cells were harvested, washed, and resuspended in a solution containing 0.5% BSA and 0.003% sodium azide in PBS. Flow cytometry analysis was carried out with a Cytomics Beckman Coulter FC 500 Cytometer. For this purpose, an area located in the low right quadrant of the FSC-SSC plot, where lymphoblast signals are expected, was gated. CFSE and RPE fluorescent signals were collected using 488nm and 635nm laser excitation wavelengths. The percentage of CD4^+^ CFSE-negative cells was registered, and no-stimuli control values were substracted to CLA- and LACK- stimulated culture aliquots.

### Quantification of IL-10 and IFN-γ in LTT supernatants

Canine IL-10 and IFN-γ DuoSet^®^ ELISA Development System (R&D Systems) kits were used following the manufacturer’s specifications. In summary, antibodies, standards and other reagents were reconstituted and/or stored as specified in the certificates of analysis, flat-bottom 96-well microplates were coated with 100μL PBS-diluted Mouse Anti-Canine IL10/IFN-γ Capture Antibody (according to the Certificate of Analysis), and the plates sealed and incubated overnight at room temperature. Then, three 400-μL washes were performed with 0.05% Tween 20 in PBS (pH 7.4), blocking with 300 μL Reagent Diluent (0.2 μm-filtered 1% BSA in PBS) was allowed at room temperature for 1h, and the washing cycles were repeated. Next, LTT supernatant samples and standards (100μL each) were added to the corresponding wells and incubated at room temperature for 2h after sealing the plate. For this purpose, standard dilutions were performed using Reagent Diluent. Then, washes were repeated and 100μL Biotinylated Mouse Anti-Canine Detection Antibody diluted with PBS in Reagent Diluent (according to the Certificate of Analysis) were added to all wells, and incubated at room temperature for 2h. Once washes were repeated, 100μL Streptavidin-HRP solution (prepared at a dilution specified in the vial using Reagent Diluent) were added to each well and incubated at room temperature in the dark for 20min. Next, washes were repeated and a 20-min room-temperature incubation in the dark with 100μL Substrate Solution (1:1 Color Reagents A and B–respectively, H_2_O_2_, and tetramethylbenzidine; R&D Systems). The reactions were stopped using 2N H_2_SO_4_. Absorbance at 450 nm was monitored with Microplate Reader 680 (BioRad) and Microplate Manager 5.2.1. software (BioRad).

## Results

### Clinical signs

**Fig 1:**
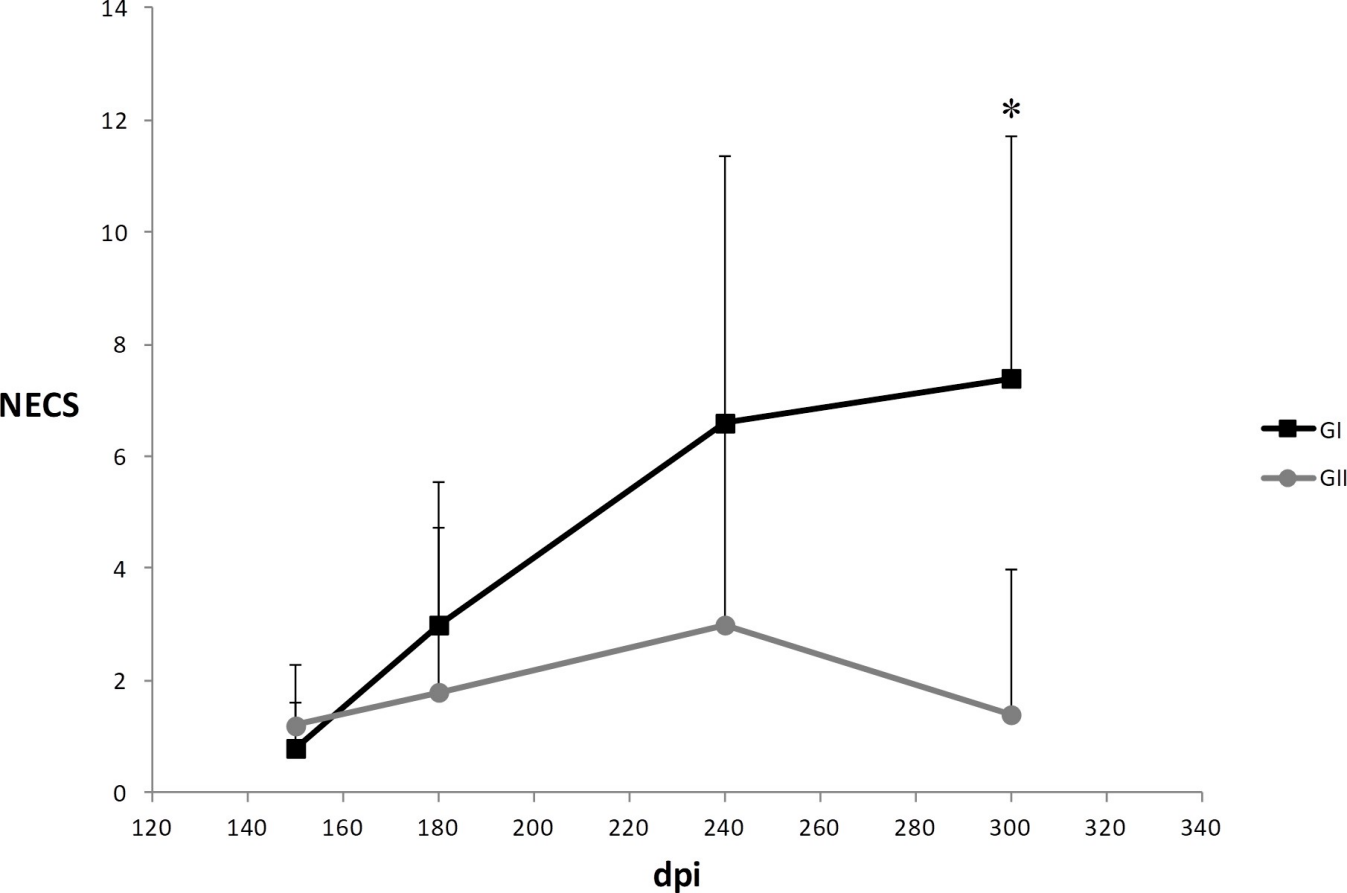
Numerical evaluation of clinical signs (NECS). Clinical signs were evaluated 150, 180, 240, and 300 dpi. GI: dogs which received two doses of 200μg pPAL-LACK + 20μg pPAL-canIL12-p35 + 20μg pPAL-canIL12-p40. GII: infection control group. Represented values are the sum of 0–5 scores provided to each of the clinical signs observed. Positive standard deviations have been represented. *Several dogs had to be euthanized before the end of the experiment (after the 240dpi time point) for ethical reasons.

Outstanding differences in clinical profiles between the control (GII) and the pPAL-LACK/pPAL-canIL12p35/pPAL-canIL12-p40-inoculated group (GI) were not observed over time (**Fig 1**). In fact, some dogs in both groups became increasingly sick over time and had to be euthanized for ethical reasons before the end of the experiment, while others did not develop any serious clinical sign associated to leishmaniasis (e.g. absence of splenomegaly but presence of mild lymphadenopathy and skin lesions). Early necropsy procedures were performed after 240 dpi. All other dogs were euthanized 300dpi as planned in the experimental design. All end time point data were processed together.

### Parasite burden

**Fig 2:**
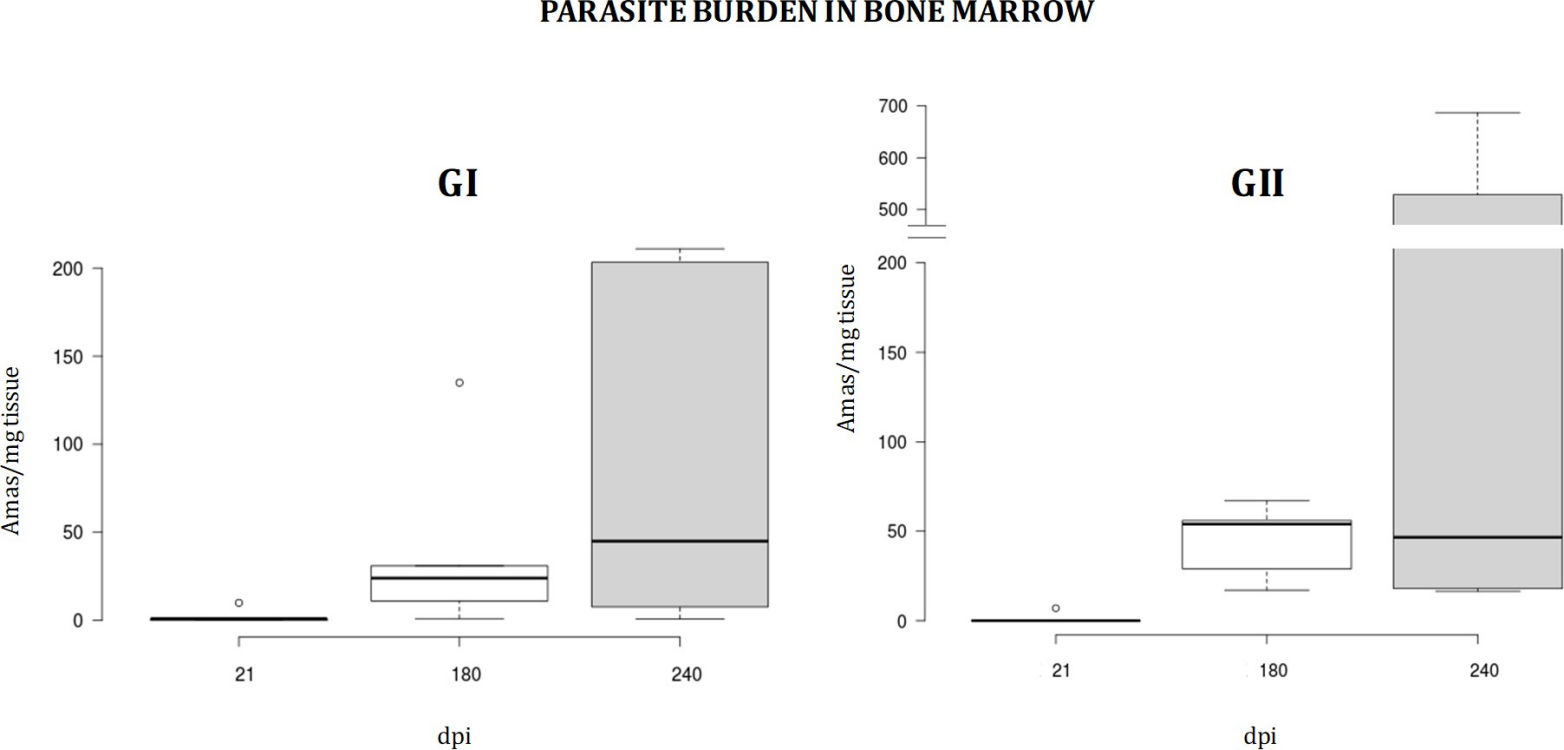
Parasite burden in bone marrow throughout the vaccination experiment. The number of amastigotes per mg tissue at 21, 180, and 240dpi is represented. The experimental infectious challenge using *L*. *infantum* promastigotes was effective. Parasite burden is higher in the infection control group (GII) than in the pPAL-LACK/pPAl-canIL12-p35/pPAL-canIL12-p40 inoculation group (GI).

**Fig 3:**
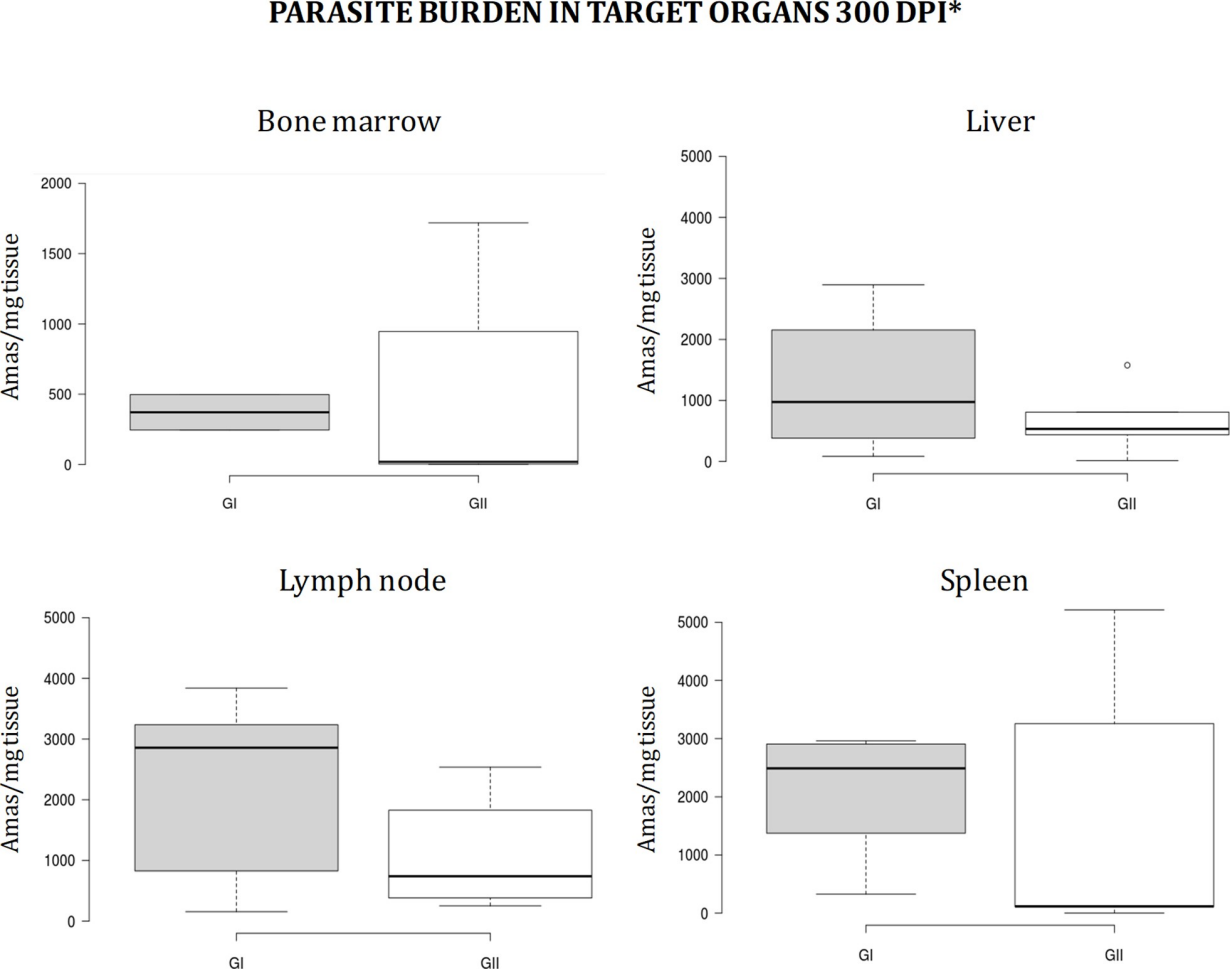
Parasite burden in target organs at the end of the experiment. Parasite burden is similar in GI (pPAL-LACK/pPAL-canIL12-p35/pPAL-canIL12-p40 group) and GII (infection control group), or higher in GI than in GII, depending on the organ. Individual variability contribute to data dispersion. *Several dogs had to be euthanized before the end of the experiment (after the 240dpi time point) for ethical reasons.

The parasite burden in bone marrow measured as number of amastigotes per mg of tissue increases in the vaccinated and inthe control group (GII) over time (**Fig 2**). These observations demonstrate that the experimental infectious challenge was effective. The median is lower in the vaccinated group than in the control group 180dpi, but is approximately equal in both groups 240 dpi, although data are quite more disperse and the maximum much higher in the control group, GII (**Fig 2**). Analysis at the end of the experiment (~300 dpi, see **Fig 3**) revealed that the parasite burden profile is reversed with respect to initial data. The parasite burden medians in bone marrow, lymph node, spleen, and liver are higher, although dispersion and maxima are wider in bone marrow and spleen (**Fig 3**). Nonetheless, differences are not statistically significant according to statistical inference using the non-parametric Mann-Whitney U test (α = 0.05), which is caused by intra-group differences between individuals. Therefore, inoculation of a primer and a booster dose of the mixture composed of 200μg pPAL-LACK, 20μg pPAL-canIL12-p35, and 20μg pPAL-canIL12-p40 constructs does not generally lead to significant reduction of parasite burden than in the challenge control group (GII). However, partial protection is observed at 180dpi, according to the considerably lower parasite burden values and range in group I.

### Humoral immune response assessment

**Fig 4:**
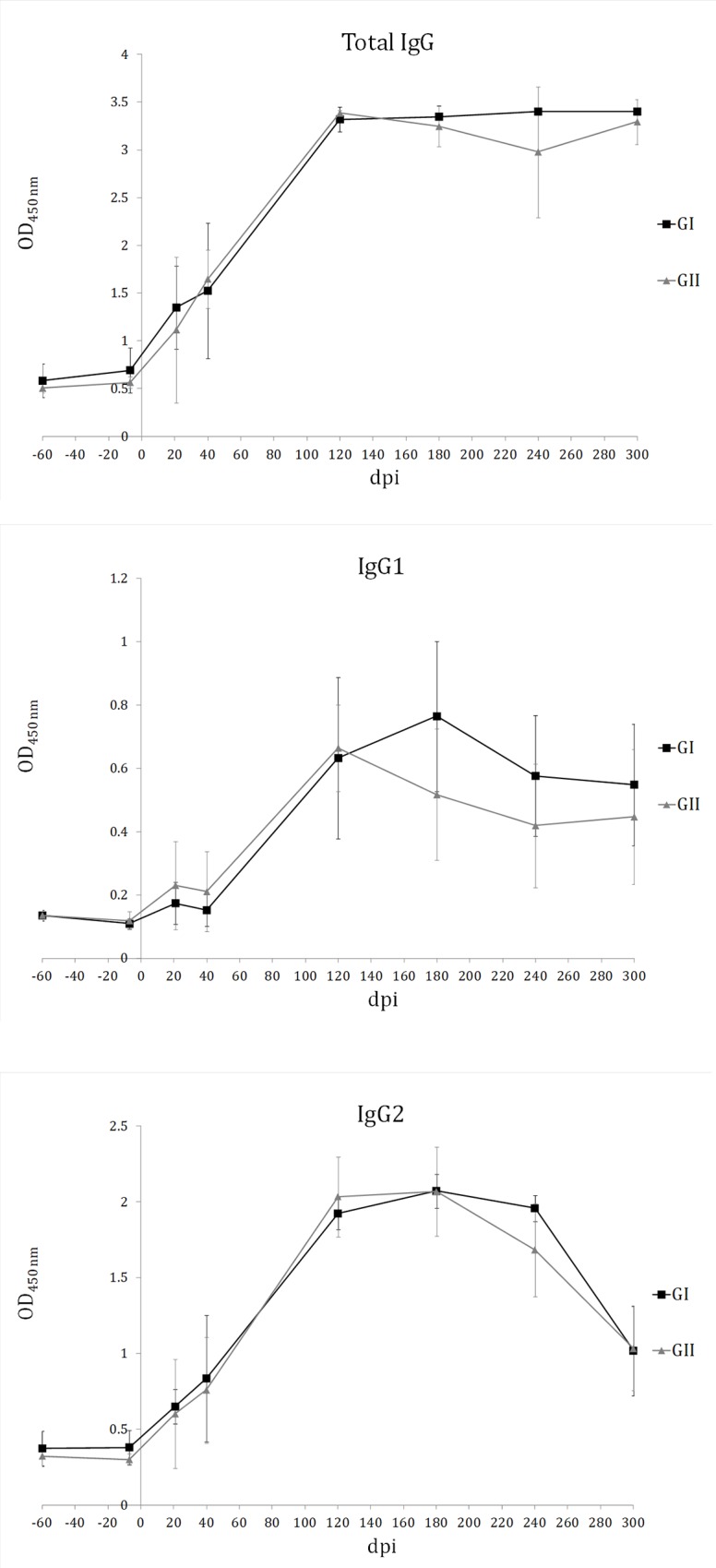
Circulating *Leishmania infantum*-specific IgG levels. IgG levels progressively increased after challenge reaching the plateau around 120dpi. No significant differences in total IgG, IgG1, and IgG2 levels are observed between GI (pPAL-LACK/pPAL-canIL12-p35/pPAL-canIL12-p40 group) and GII (infection control group). This is additional evidence for experimental infectious challenge efficacy in both groups. Immunity to *Leishmania* spp. is determined by cellular immune responses.

*Leishmania*-specific IgG1, IgG2, and total IgG level follow-up throughout the experiment revealed absence of *Leishmania*-specific antibodies at the beginning of the experiment and a gradual increase after challenge, reaching the plateau around 120dpi (**Fig 4**). These are additional data demonstrating the effectiveness of the experimental infectious challenge. No statistically significant differences (Mann-Whitney U test, α = 0.05) in the IgG isotype circulating levels were observed between the experimental (GI) and the control (GII) group, including total IgG and IgG1 and 2 subclasses.

### Cellular immune response analysis

**Fig 5:**
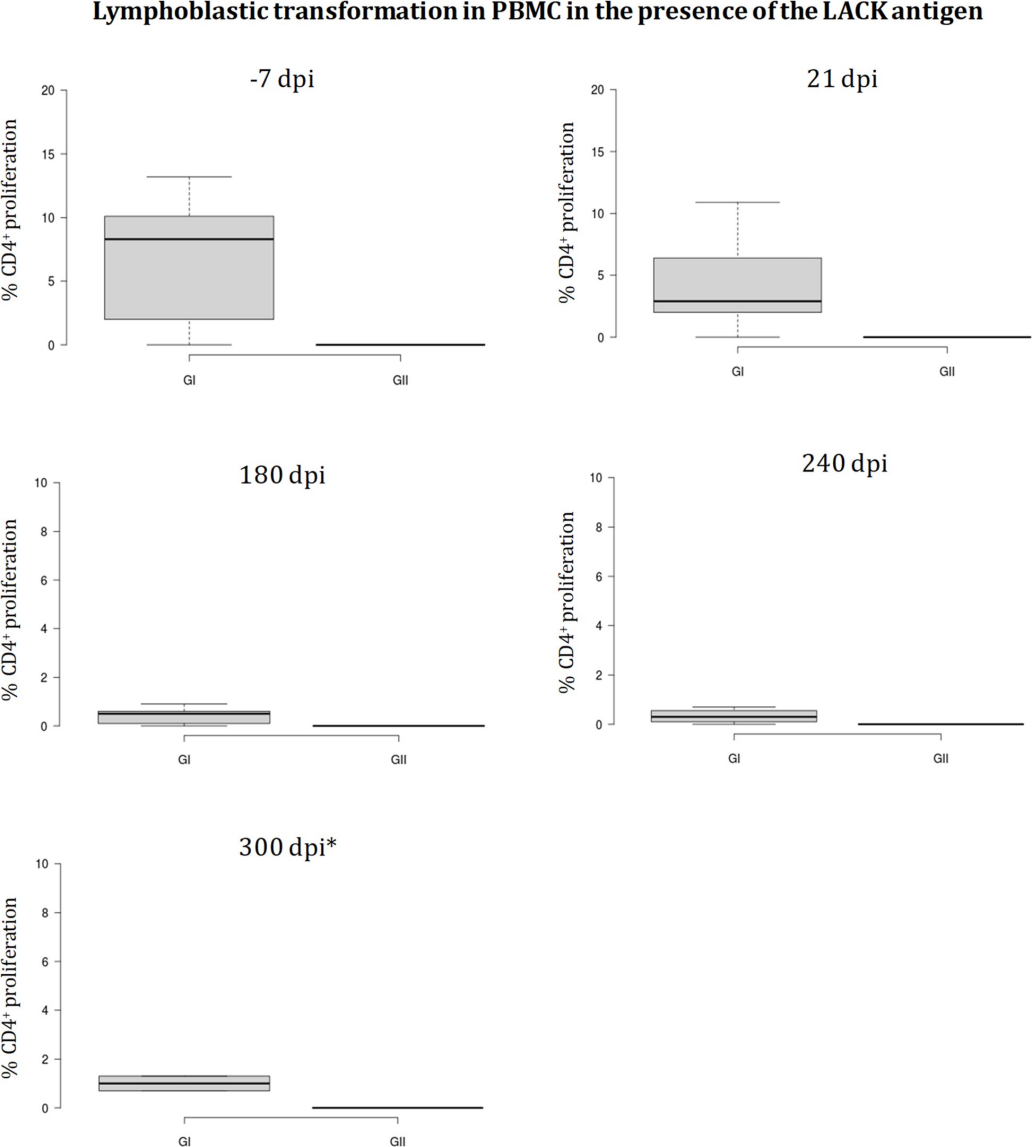
Lymphoblastic transformation test (LTT). CD4+-proliferation against the LACK protein is observed only in group GI (pPAL-LACK/pPAL-canIL12-p35/pPAL-canIL12-p40). Proliferation decreases 180, 240, and 300dpi. *Several dogs had to be euthanized before the end of the experiment (after the 240dpi time point) for ethical reasons.

Lymphoblastic transformation of PBMC in the presence of recombinant LACK protein is observed -7dpi (53 days after prime inoculation and 38 days after administration of the boost dose) in pPAL-LACK/pPAL-canIL12p35/pPAL-canIL12-p40-inoculated dogs (group I), but not in the control group, GII (**Fig 5**). The proliferation median is ~8%, which decreases to ~3% shortly after challenge (21dpi), and is maintained around ~1% until the end of the experiment. No CD4^+^ proliferation in the presence of the LACK protein is observed in group II. The differences between both groups are statistically significant (Mann-Whitney U test, α = 0.05).

**Fig 6:**
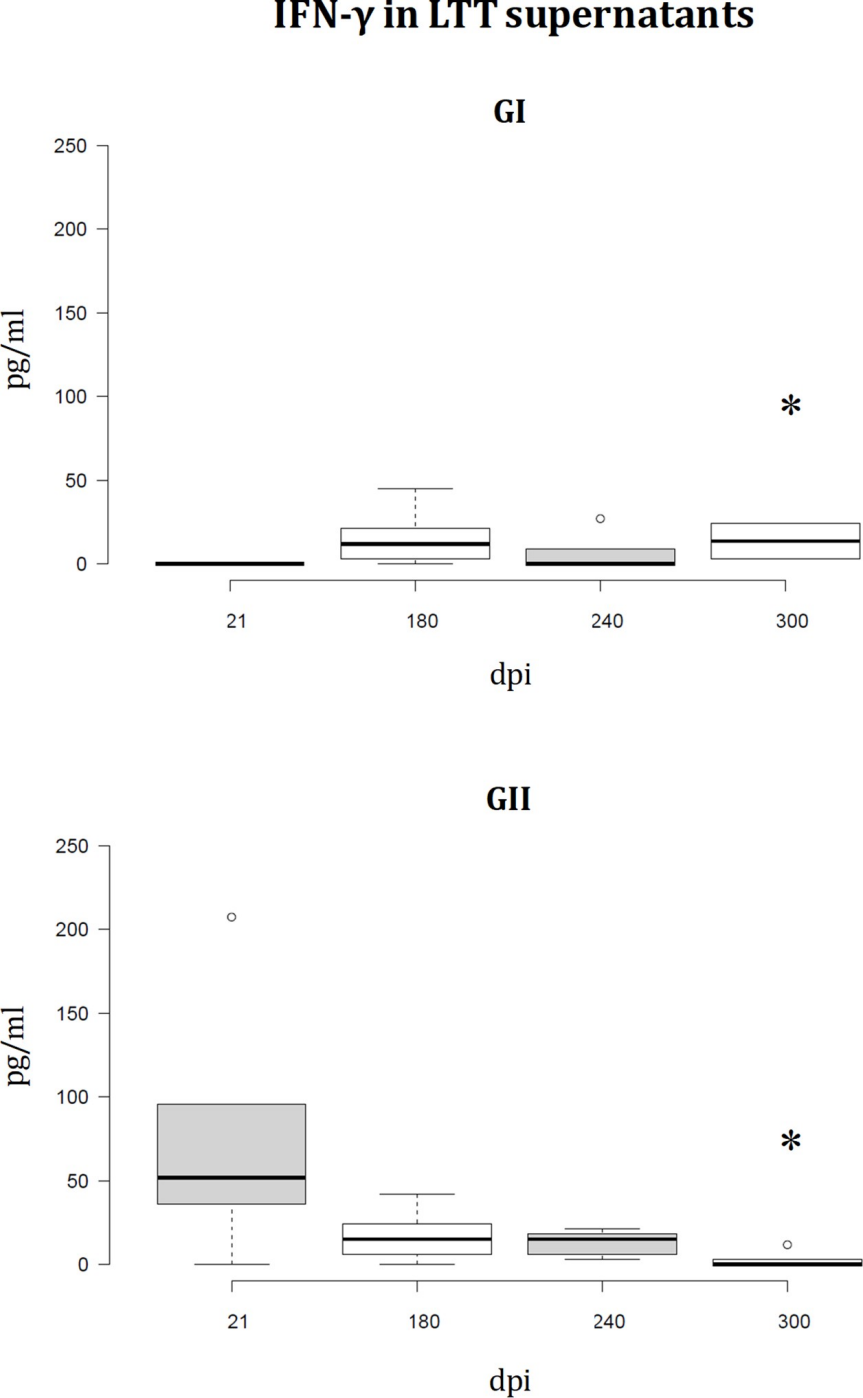
IFN-γ concentration in LTT supernatants. A specific commercially available ELISA procedure was used to determine IFN-γ concentration (pg/mL) in LTT supernatants, which had been immediately stored at -20°C after separation from cells. IFN-γ fluctuates in LTT supernatants from GI dogs (pPAL-LACK/pPAL-canIL12-p35/pPAL-canIL12-p40), and gradually decrease in the control group (GII). At the beginning (21dpi), IFN-γ is observed only in GII. *Several dogs had to be euthanized before the end of the experiment (after the 240dpi time point) for ethical reasons.

**Fig 7:**
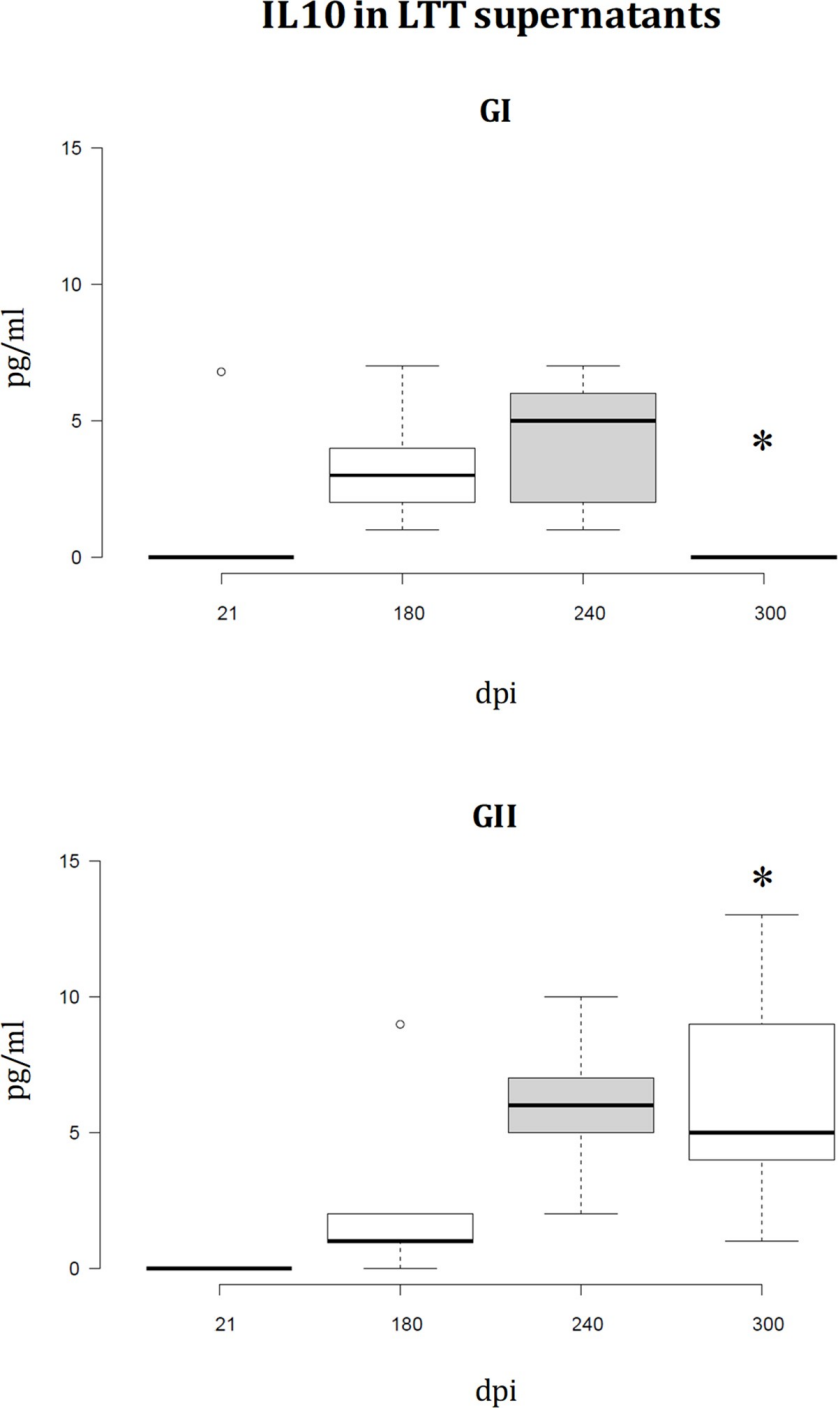
IL10 levels in LTT supernatants. A specific commercially available ELISA procedure was used to determine IL10 concentration (pg/mL) in LTT supernatants, which had been immediately stored at -20°C after separation from cells. The IL10 peak is observed earlier in GI (pPAL-LACK/pPAL-canIL12-p35/pPAL-canIL12-p40) than in GII (infection control group). *Several dogs had to be euthanized before the end of the experiment (after the 240dpi time point) for ethical reasons.

Cytokine profile analysis of LTT supernatants has revealed fluctuations in the IFN-γ and IL10 levels of proliferating PBMC in both groups (**Figs 6** and **7**). Two IFN-γ peaks are observed in group I. The second peak is simultaneous to the decrease in group II. Conversely, group II shows relatively high IFN-γ levels at the beginning of the experiment compared to the almost inexistent levels in group I (21dpi), which turn out to be approximately equal in both groups 120dpi (**Fig 6**). There is an IL10 peak in both groups, which was observed earlier in group I (**Fig 7**).

## Discussion

The LACK antigen gene administered in a mammalian expression plasmid vehicle is able to partially protect mice against infection by *L*. *major* (cutaneous signs) [30,31]. This was also observed in dogs against *L*. *infantum* infection (cutaneous and visceral signs) and was improved using a recombinant plasmid in the prime dose and a recombinant vaccinia virus in the booster dose by the subcutaneous route [27,33,34]. The intranasal administration route was shown to be more effective than the subcutaneous route in the murine model when administering naked DNA [35]. More recently, we have improved safety using the antibiotic resistance-free plasmid pPAL as the LACK gene vehicle by the intranasal route [26]. This safety improvent has two contributions. First, antibiotic resistance genes were removed. Second, the *E*. *coli fab I* gene used to replace them as a selection marker contains a long CpG island which hypothetically acts as an adjuvant. In fact, pPAL-LACK prime-boost vaccination administered by the intranasal route approximately protects as well as an heterologous regime which consists of using the same plasmid as the primer and the recombinant modified vaccinia virus Ankara strain (MVA-LACK) as the booster [26]. Using the dog model for studying *L*. *infantum* infection and for vaccination trials is challenging for a variety of reasons. Some are related with lodging, animal care, and suffering amelioration, complying with animal experimentation regulations. Others are related with genetic variability and reduced sample number intrinsic to experimentation. However, using this model is essential because results in more conventional laboratory animal models cannot usually be extrapolated to the canine model, which acts as the natural reservoir in the parasite's life cycle. An example is the Th1/Th2 response dichotomy, which is observed in mice but not in dogs [20,23,24,25]. Even though these difficulties are intrinsic to canine leishmaniasis vaccine trials, significant differences between vaccinated and control groups were always observed using LACK gene constructs in our hands.

IL12 is a proinflammatory cytokine which stimulates cytotoxic T lymphocytes and NK cells, as well as T helper type 1 (Th1) lymphocytes, leading to IFN-γ production. IL12 is able to enhance lymphocyte proliferation and IFN-γ production in *L*. *infantum* infected dogs, which may be an alternative therapy strategy because a protective Th1 response is favoured [28]. In an attempt to improve efficacy of 200μg pPAL-LACK prime/boost inoculations by the intranasal route, we co-administered pPAL-canIL12-p35 and pPAL-canIL12-p40 plasmid constructs (20μg each). The experimental infectious challenge was successful because all dogs became positive to *L*. *infantum* over time according to qPCR-based parasite burden analysis and *Leishmania*-specific IgG production (**Figs 2–4**). Some dogs became sick in both groups and had to be euthanized before the end of the experiment, while others did not show serious clinical signs associated to leishmaniasis. Early necropsy procedures were performed after 240 dpi. This circumstance has been indicated with an asterisk in **Figs 1, 3, 4** and **7** (data in **S1 Table**). All other dogs were euthanized 300dpi as planned in the experimental design. All end time point data were processed together. Differences in disease progression within groups are presumably due to aforementioned individual variability. This causes the lack of statistical significance of the differences observed between groups, as explained next. Despite pPAL-LACK protects [26], no differences in clinical profiles are globally observed when genes encoding for IL12 subunits are co-administered (**Fig 1**). Parasite burden is considerably higher in the control group (GII) than in the experimental group (GI) 240 dpi (**Fig 2**). A different picture is observed at the end of the experiment (300 dpi or earlier, as already mentioned): even though dispersion is higher in the control group (GII) in two out of four target organs, medians are lower in all of them (**Fig 3**). In summary, clinical profile follow-up and parasite burden analysis lead to conclude that no clear protection against the parasite is observed in the experimental group (GI) compared to the control group (GII).

Similar IgG titles in groups I and II (**Fig 4**) are not surprising because the parasite is intracellular and consequently, a cellular immune response is required for parasite clearance. In fact, studies showing protection conferred by the LACK gene presented similar humoral immune response results [26]. pPAL-LACK/pPAL-canIL12-p35/pPAL-canIL12-p40 cocktail inoculation was able to induce primary CD4^+^ activation which decreases over time, as a difference with the control group, GII (**Fig 5**). This finding suggests that the pPAL-LACK construct initially triggers T helper response activation. IFN-γ and IL10 levels fluctuate in both groups as observed in previous experiments [26,27]. In this case, an IFN-γ early peak after infection is observed only in the control group (GII); next, fluctuations alternate between both groups (**Fig 6**). Conversely, an IL10 peak occurs earlier in the experimental group, GI (**Fig 7**). In principle, IL10 is a typical Th2 cytokine, whereas IFN-γ is related to a Th1 response. The Th1/Th2 protection/susceptibility dichotomy has been demonstrated in the experimental murine model against *L*. *major* infection (reviewed in [20]). However, fluctuations were observed in previous experiments using only the LACK gene [26,27] and in this study co-administering IL12 subunit genes. This is in agreement with a balanced Th1/Th2 response in the canine model as the hypothetical key event leading to protection. Such balanced response occurs when there is no relevant IFN-γ or IL10 excess [23,24,25]. As cytokine fluctuations have been observed in both groups configuring this experiment (**Figs 6** and **7**) and is not a distinctive feature compared to previous experiments [26,27], we infer that this is not the factor causing IL12 failure as an adjuvant in this case. A recombinant protein preparation including IL12 and a mixture of cysteine peptidases did not protect dogs against *L*. *infantum* either [43]. Therefore, vaccine type does not seem to be the cause of failure. We propose two possible causes of pPAL-canIL12-p35 and pPAL-canIL12-p40 constructs acting as inhibitors of protection conferred by the pPAL-LACK vaccine: i) IL12 is a good cytokine therapy candidate [28] but may not function as a Th1 immune response enhancer in uninfected dogs; and ii) amounts of IL12 subunit genes administered to dogs by the intranasal route may not be adequate and may cause unexpected immune response de-regulation counteracting pPAL-LACK vaccine protection effects. In any case, using pPAL-LACK vaccine alone has led to much more promising results in canine leishmaniasis vaccination trials [26], as well as heterologous regimes using recombinant vaccinia viruses [27,34]. Poot et al. also hypothesized that dose and timing may be important for IL12 administration [43]. In view of all discussed data, future trials trying to optimize amounts of IL12 subunit-encoding genes would not lead to successful results improving pPAL-LACK protection in our opinion. This would probably cause worthless use of a lot expenses in human and material resources, as well as outstanding use of Beagle dogs, breaking the reduction principle in animal experimentation. This observation should not be generalized for other vaccine candidates against the same parasite or other pathogens. Comparing results obtained using an antigen without and with IL12 would be useful for case-by-case risk-benefit prediction of future IL12 dose and timing optimization experiments.

Finally, it is important to remark that the experiment presented herein is an example of the substantial differences in biology and pathology between *Leishmania* species and parasite-host pairs (fundamentally, *L*. *major*-mice and *L*. *infantum*-dogs). In fact, successful adjuvant effects of IL12 observed in protection experiments against *L*. *major* infection in mice has not been observed in dogs until now. Another example of lack of correspondence between models is Th1/Th2 response dichotomy, which has been clearly observed only in mice.

## Supporting information

S1 Fig pPAL-IL12-p35 and pPAL-IL12-p40. Plasmid maps were generated using PlasMapper software. ORF1 is the respective p35 or p40 subunit-encoding ORF.
(PPTX)

S2 Fig pPAL-canIL12-p35 and pPAL-canIL12-p40 sequences. Restriction sites used for cloning in the pPAL vector: SalI (GTCGAC), MboI (ACGCGT), XbaI (TCTAGA). Color legend: green, canIL12p35 ORF; blue, canIL12p40 ORF; red, fabIgene.
(DOCX)

S1 Table Supporting data. (XLSX)
