# Supplementary material for: IL12 p35 and p40 subunit genes administered as pPAL plasmid constructs do not improve protection of pPAL-LACK vaccine against canine leishmaniasis
Source: PLoS One. 2019 Feb 22;14(2):e0212136. doi: 10.1371/journal.pone.0212136 (PMC6386296; doi:10.1371/journal.pone.0212136)
Supplement: S1 Fig — Plasmid maps were generated using PlasMapper software. ORF1 is the respective p35 or p40 subunit-encoding ORF. (PPTX) [file pone.0212136.s001.pptx]

## Slide 1
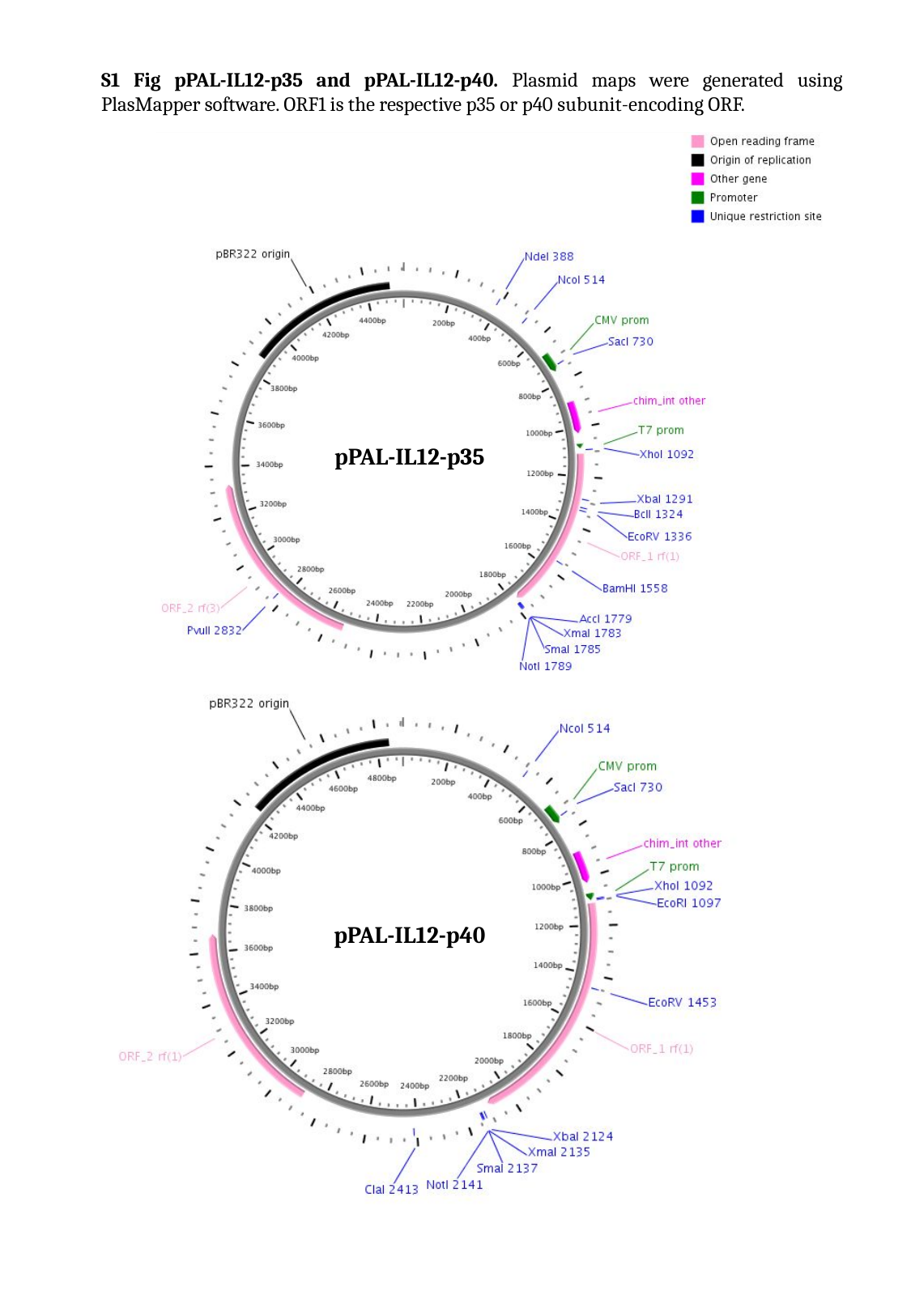

S1 Fig pPAL-IL12-p35 and pPAL-IL12-p40. Plasmid maps were generated using PlasMapper software. ORF1 is the respective p35 or p40 subunit-encoding ORF.
pPAL-IL12-p35
pPAL-IL12-p40
