# Supplementary material for: IL12 p35 and p40 subunit genes administered as pPAL plasmid constructs do not improve protection of pPAL-LACK vaccine against canine leishmaniasis
Source: PLoS One. 2019 Feb 22;14(2):e0212136. doi: 10.1371/journal.pone.0212136 (PMC6386296; doi:10.1371/journal.pone.0212136)
Supplement: S2 Fig — Restriction sites used for cloning in the pPAL vector: SalI (GTCGAC), MboI (ACGCGT), XbaI (TCTAGA). Color legend: green, canIL12p35 ORF; blue, canIL12p40 ORF; red, fabIgene. (DOCX) [file pone.0212136.s002.docx]

**S2 Fig. pPAL-canIL12-p35 and pPAL-canIL12-p40 sequences.** Restriction sites used for cloning in the pPAL vector: SalI (GTCGAC), MboI (ACGCGT), XbaI (TCTAGA). Color legend: canIL12p35 ORF; canIL12p40 ORF; fabIgene.

**pPAL-canIL12-p35**

tcaatattggccattagccatattattcattggttatatagcataaatcaatattggctattggccattgcatacgttgtatctatatcataatatgtacatttatattggctcatgtccaatatgaccgccatgttggcattgattattgactagttattaatagtaatcaattacggggtcattagttcatagcccatatatggagttccgcgttacataacttacggtaaatggcccgcctggctgaccgcccaacgacccccgcccattgacgtcaataatgacgtatgttcccatagtaacgccaatagggactttccattgacgtcaatgggtggagtatttacggtaaactgcccacttggcagtacatcaagtgtatcatatgccaagtccgccccctattgacgtcaatgacggtaaatggcccgcctggcattatgcccagtacatgaccttacgggactttcctacttggcagtacatctacgtattagtcatcgctattaccatggtgatgcggttttggcagtacaccaatgggcgtggatagcggtttgactcacggggatttccaagtctccaccccattgacgtcaatgggagtttgttttggcaccaaaatcaacgggactttccaaaatgtcgtaacaactgcgatcgcccgccccgttgacgcaaatgggcggtaggcgtgtacggtgggaggtctatataagcagagctcgtttagtgaaccgtcagatcactagaagctttattgcggtagtttatcacagttaaattgctaacgcagtcagtgcttctgacacaacagtctcgaacttaagctgcagtgactctcttaaggtagccttgcagaagttggtcgtgaggcactgggcaggtaagtatcaaggttacaagacaggtttaaggagaccaatagaaactgggcttgtcgagacagagaagactcttgcgtttctgataggcacctattggtcttactgacatccactttgcctttctctccacaggtgtccactcccagttcaattacagctcttaaggctagagtacttaatacgactcactataggctagcctcgagaattc**ACGCGT**atgtgcccgccgcgcggcctcctccttgtgaccatcctggtcctgctaagccacctggaccaccttacttgggccaggagcctccccacagcctcaccgagcccaggaatattccagtgcctcaaccactcccaaaacctgctgagagccgtcagcaacacgcttcagaaggccagacaaactctagattatattccctgcacttccgaagagattgatcatgaagatatcacaaaggataaaaccagcacagtggaggcctgcttaccactggaattaaccatgaatgagagttgcctggcttccagagagatctctttgataactaacgggagttgcctggcctctggaaaggcctcttttatgacggtcctgtgccttagcagcatctatgaggacttgaagatgtaccagatggaattcaaggccatgaacgcaaagcttttaatggatcccaagaggcagatctttctggatcaaaacatgttgacagctatcgatgagctgttacaggccctgaatttcaacagtgtgactgtgccacagaaatcctcccttgaagagccggatttttataaaactaaaatcaagctctgcatacttcttcatgctttcagaattcgtgcggtgaccatcgatagaatgatgagttatctgaattcttcctaa**GTCGA**Cccgggcggccgcttccctttagtgagggttaatgcttcgagcagacatgataagatacattgatgagtttggacaaaccacaactagaatgcagtgaaaaaaatgctttatttgtgaaatttgtgatgctattgctttatttgtaaccattataagctgcaataaacaagttaacaacaacaattgcattcattttatgtttcaggttcagggggagatgtgggaggttttttaaagcaagtaaaacctctacaaatgtggtaaaatccgataaggatcgatccgggctggcgtaatag*cgaagaggcccgcaccgatcgcccttcccaacagttgcgcagcctgaatggcgaatggacgcgccctgtagcggcgcattaagcgcggcgggtgtggtggttacgcgcagcgtgaccgctacacttgccagcgttaattaagtgctggagaatattcggcaaggtctgaaccgtcccagccatcgccatgaaagggttaggggctgtatgagcctgtttgttgctggggtaacaatatttgcacaatacggtcccctcgcccctctggggagagggttagggtgaggggaaaagcgccccccctgccgcagcctgctccggtcggacctggcaactatagctactcacagccaggttgattataataaccgtttatctgttcgtactgtttactaaaacgacgaatcgcctgattttcaggcacaacaagcatcaacaataaggattaaagctatgggttttctttccggtaagcgcattctggtaaccggtgttgccagcaaactatccatcgcctacggtatcgctcaggcgatgcaccgcgaaggagctgaactggcattcacctaccagaacgacaaactgaaaggccgcgtagaagaatttgccgctcaattgggttctgacatcgttctgcagtgcgatgttgcagaagatgccagcatcgacaccatgttcgctgaactggggaaagtttggccgaaatttgacggtttcgtacactctattggttttgcacctggcgatcagctggatggtgactatgttaacgccgttacccgtgaaggcttcaaaattgcccacgacatcagctcctacagcttcgttgcaatggcaaaagcttgccgctccatgctgaatccgggttctgccctgctgaccctttcctaccttggcgctgagcgcgctatcccgaactacaacgttatgggtctggcaaaagcgtctctggaagcgaacgtgcgctatatggcgaacgcgatgggtccggaaggtgtgcgtgttaacgccatctctgctggtccgatccgtactctggcggcttccggtatcaaagacttccgcaaaatgctggctcattgcgaagccgttaccccgattcgccgtaccgttactattgaagatgtgggtaactctgcggcattcctgtgctccgatctctctgccggtatctccggtgaagtagtccacgttgacggcggtttcagcatcgctgcaatgaacgaactcgaactgaaataattaattaaccgcgtatggtgcactctcagtacaatctgctctgatgccgcatagttaagccagccccgacacccgccaacacccgctgacgcgccctgacgggcttgtctgctcccggcatccgcttacagacaagctgtgaccgtctccgggagctgcatgtgtcagaggttttcacaagttgcaggaccacttctgcgctcggcccttccggctggctggtttattgctgataaatctggagccggtgagcgtgggtctcgcggtatcattgcagcactggggccagatggtaagccctcccgtatcgtagttatctacacgacggggagtcaggcaactatggatgaacgaaatagacagatcgctgagataggtgcctcactgattaagcattggtaactgtcagaccaagtttactcatatatactttagattgatttaaaacttcatttttaatttaaaaggatctaggtgaagatcctttttgataatctcatgaccaaaatcccttaacgtgagttttcgttccactgagcgtcagaccccgtagaaaagatcaaaggatcttcttgagatcctttttttctgcgcgtaatctgctgcttgcaaacaaaaaaaccaccgctaccagcggtggtttgtttgccggatcaagagctaccaactctttttccgaaggtaactggcttcagcagagcgcagataccaaatactgtccttctagtgtagccgtagttaggccaccacttcaagaactctgtagcaccgcctacatacctcgctctgctaatcctgttaccagtggctgctgccagtggcgataagtcgtgtcttaccgggttggactcaagacgatagttaccggataaggcgcagcggtcgggctgaacggggggttcgtgcacacagcccagcttggagcgaacgacctacaccgaactgagatacctacagcgtgagctatgagaaagcgccacgcttcccgaagggagaaaggcggacaggtatccggtaagcggcagggtcggaacaggagagcgcacgagggagcttccagggggaaacgcctggtatctttatagtcctgtcgggtttcgccacctctgacttgagcgtcgatttttgtgatgctcgtcaggggggcggagcctatggaaaaacgccagcaacgcggcctttttacggttcctggccttttgctggccttttgctcacatggctcgacagatct

**pPAL-canIL12p40**

tcaatattggccattagccatattattcattggttatatagcataaatcaatattggctattggccattgcatacgttgtatctatatcataatatgtacatttatattggctcatgtccaatatgaccgccatgttggcattgattattgactagttattaatagtaatcaattacggggtcattagttcatagcccatatatggagttccgcgttacataacttacggtaaatggcccgcctggctgaccgcccaacgacccccgcccattgacgtcaataatgacgtatgttcccatagtaacgccaatagggactttccattgacgtcaatgggtggagtatttacggtaaactgcccacttggcagtacatcaagtgtatcatatgccaagtccgccccctattgacgtcaatgacggtaaatggcccgcctggcattatgcccagtacatgaccttacgggactttcctacttggcagtacatctacgtattagtcatcgctattaccatggtgatgcggttttggcagtacaccaatgggcgtggatagcggtttgactcacggggatttccaagtctccaccccattgacgtcaatgggagtttgttttggcaccaaaatcaacgggactttccaaaatgtcgtaacaactgcgatcgcccgccccgttgacgcaaatgggcggtaggcgtgtacggtgggaggtctatataagcagagctcgtttagtgaaccgtcagatcactagaagctttattgcggtagtttatcacagttaaattgctaacgcagtcagtgcttctgacacaacagtctcgaacttaagctgcagtgactctcttaaggtagccttgcagaagttggtcgtgaggcactgggcaggtaagtatcaaggttacaagacaggtttaaggagaccaatagaaactgggcttgtcgagacagagaagactcttgcgtttctgataggcacctattggtcttactgacatccactttgcctttctctccacaggtgtccactcccagttcaattacagctcttaaggctagagtacttaatacgactcactataggctagcctcgagaattc**ACGCGT**atgcatcctcagcagttggtcatctcctggttttccctcgttttgctggcgtcttccctcatgaccatatgggaactggagaaagatgtttatgttgtagagttggactggcaccctgatgcccccggagaaatggtggtcctcacctgccatacccctgaagaagatgacatcacttggacctcagcgcagagcagtgaagtcctaggttctggtaaaactctgaccatccaagtcaaagaatttggagatgctggccagtatacctgccataaaggaggcaaggttctgagccgctcactcctgttgattcacaaaaaagaagatggaatttggtccactgatatcttaaaggaacagaaagaatccaaaaataagatctttctgaaatgtgaggcaaagaattattctggacgtttcacatgctggtggctgacggcaatcagtactgatttgaaattcagtgtcaaaagtagcagaggcttctctgacccccaaggggtgacatgtggagcagtgacactttcagcagagagggtcagagtggacaacagggattataagaagtacacagtggagtgtcaggaaggcagtgcctgcccctctgccgaggagagcctacccatcgaggtcgtggtggatgctattcacaagctcaagtatgaaaactacaccagcagcttcttcatcagagacatcatcaaaccagacccacccacaaacctgcagctgaagccattgaaaaattctcggcacgtggaggtcagctgggaataccccgacacctggagcaccccacattcctacttctccctgacattttgcgtacaggcccagggcaagaacaatagagaaaagaaagatagactctgcgtggacaagacctcagccaaggtcgtgtgccacaaggatgccaagatccgcgtgcaagcccgagaccgctactatagttcatcctggagcgactgggcatctgtgtcctgcagttaggttccacccccaggatgaatcttgg**TCTAGA**gtcgacccgggcggccgcttccctttagtgagggttaatgcttcgagcagacatgataagatacattgatgagtttggacaaaccacaactagaatgcagtgaaaaaaatgctttatttgtgaaatttgtgatgctattgctttatttgtaaccattataagctgcaataaacaagttaacaacaacaattgcattcattttatgtttcaggttcagggggagatgtgggaggttttttaaagcaagtaaaacctctacaaatgtggtaaaatccgataaggatcgatccgggctggcgtaatag*cgaagaggcccgcaccgatcgcccttcccaacagttgcgcagcctgaatggcgaatggacgcgccctgtagcggcgcattaagcgcggcgggtgtggtggttacgcgcagcgtgaccgctacacttgccagcgttaattaagtgctggagaatattcggcaaggtctgaaccgtcccagccatcgccatgaaagggttaggggctgtatgagcctgtttgttgctggggtaacaatatttgcacaatacggtcccctcgcccctctggggagagggttagggtgaggggaaaagcgccccccctgccgcagcctgctccggtcggacctggcaactatagctactcacagccaggttgattataataaccgtttatctgttcgtactgtttactaaaacgacgaatcgcctgattttcaggcacaacaagcatcaacaataaggattaaagctatgggttttctttccggtaagcgcattctggtaaccggtgttgccagcaaactatccatcgcctacggtatcgctcaggcgatgcaccgcgaaggagctgaactggcattcacctaccagaacgacaaactgaaaggccgcgtagaagaatttgccgctcaattgggttctgacatcgttctgcagtgcgatgttgcagaagatgccagcatcgacaccatgttcgctgaactggggaaagtttggccgaaatttgacggtttcgtacactctattggttttgcacctggcgatcagctggatggtgactatgttaacgccgttacccgtgaaggcttcaaaattgcccacgacatcagctcctacagcttcgttgcaatggcaaaagcttgccgctccatgctgaatccgggttctgccctgctgaccctttcctaccttggcgctgagcgcgctatcccgaactacaacgttatgggtctggcaaaagcgtctctggaagcgaacgtgcgctatatggcgaacgcgatgggtccggaaggtgtgcgtgttaacgccatctctgctggtccgatccgtactctggcggcttccggtatcaaagacttccgcaaaatgctggctcattgcgaagccgttaccccgattcgccgtaccgttactattgaagatgtgggtaactctgcggcattcctgtgctccgatctctctgccggtatctccggtgaagtagtccacgttgacggcggtttcagcatcgctgcaatgaacgaactcgaactgaaataattaattaaccgcgtatggtgcactctcagtacaatctgctctgatgccgcatagttaagccagccccgacacccgccaacacccgctgacgcgccctgacgggcttgtctgctcccggcatccgcttacagacaagctgtgaccgtctccgggagctgcatgtgtcagaggttttcacaagttgcaggaccacttctgcgctcggcccttccggctggctggtttattgctgataaatctggagccggtgagcgtgggtctcgcggtatcattgcagcactggggccagatggtaagccctcccgtatcgtagttatctacacgacggggagtcaggcaactatggatgaacgaaatagacagatcgctgagataggtgcctcactgattaagcattggtaactgtcagaccaagtttactcatatatactttagattgatttaaaacttcatttttaatttaaaaggatctaggtgaagatcctttttgataatctcatgaccaaaatcccttaacgtgagttttcgttccactgagcgtcagaccccgtagaaaagatcaaaggatcttcttgagatcctttttttctgcgcgtaatctgctgcttgcaaacaaaaaaaccaccgctaccagcggtggtttgtttgccggatcaagagctaccaactctttttccgaaggtaactggcttcagcagagcgcagataccaaatactgtccttctagtgtagccgtagttaggccaccacttcaagaactctgtagcaccgcctacatacctcgctctgctaatcctgttaccagtggctgctgccagtggcgataagtcgtgtcttaccgggttggactcaagacgatagttaccggataaggcgcagcggtcgggctgaacggggggttcgtgcacacagcccagcttggagcgaacgacctacaccgaactgagatacctacagcgtgagctatgagaaagcgccacgcttcccgaagggagaaaggcggacaggtatccggtaagcggcagggtcggaacaggagagcgcacgagggagcttccagggggaaacgcctggtatctttatagtcctgtcgggtttcgccacctctgacttgagcgtcgatttttgtgatgctcgtcaggggggcggagcctatggaaaaacgccagcaacgcggcctttttacggttcctggccttttgctggccttttgctcacatggctcgacagatct
